# Hyperchloremia is associated with 30-day mortality in major trauma patients: a retrospective observational study

**DOI:** 10.1186/s13049-016-0311-7

**Published:** 2016-10-04

**Authors:** Jin Young Lee, Tae Hwa Hong, Kyung Won Lee, Myung Jae Jung, Jae Gil Lee, Seung Hwan Lee

**Affiliations:** 1 Division of Critical Care and Trauma Surgery, Department of Surgery, Yonsei University College of Medicine, 50-1 Yonsei-ro, Seodaemun-gu, Seoul, 03722 Republic of Korea; 2 Trauma Training Center, Severance Hospital, Yonsei University Health System, Seoul, Republic of Korea

**Keywords:** Chloride, Mortality, Trauma, Normal saline, Resuscitation

## Abstract

**Background:**

Chloride is important for maintaining acid-base balance, muscular activity, osmosis and immunomodulation. In patients with major trauma, chloride levels increase after fluid therapy; this is associated with poor clinical outcomes. The purpose of this study was to determine whether hyperchloremia was associated with increased mortality in patients who had sustained major trauma.

**Methods:**

This study enrolled 266 major trauma patients by retrospective chart review, from January 2011 to December 2015. Patients were older than 16 years; were admitted to an intensive care unit; survived more than 48 h; and had sustained major trauma, defined as an injury severity score ≥ 16. Hyperchloremia was defined as a chloride level > 110mEq/L. Delta chloride (Δchloride) was defined as the difference between the serum chloride level measured 48-h post-admission and the initial level. Clinical and laboratory variables were compared between survivors (*n* = 235) and non-survivors (*n* = 31). A multivariate logistic regression analysis was performed to assess the association between hyperchloremia 48-h post-admission (hyperchloremia-48) and 30-day mortality.

**Results:**

The overall 30-day mortality was 11.7 % (*n* = 31). Hyperchloremia-48 occurred in 65 patients (24.4 %) and the incidence was significantly different between survivors and non-survivors (19.6 vs. 61.3 %, respectively, *p* < 0.001). Multivariate logistic analysis identified hyperchloremia-48 and Δchloride as independent predictive factors for 30-day mortality in major trauma patients.

**Discussion:**

Infusion of chloride-rich solutions, such as normal saline, is itself associated with hyperchloremia, which has been associated with poor patient outcomes. Patients receiving normal saline were more likely to suffer major postoperative complications, acute kidney injury, and infections. Moreover, large changes in serum chloride levels correlated with greater in-hospital mortality.

**Conclusion:**

Hyperchloremia 48-h post-admission and Δchloride was associated with 30-day mortality in major trauma patients. These indices may be useful prognostic markers.

## Background

Chloride is the major anion in blood, accounting for approximately one-third of plasma tonicity, for 97–98 % of all strong anionic charges, and for two-thirds of all negative charges in plasma [1]. It plays a pivotal role in many body functions, including acid-base balance, muscular activity, osmosis and immunomodulation [2]. Despite its physiologic importance, chloride has received much less attention than other routinely measured electrolytes [1]. However, the more our understanding of acid-base and chloride channel physiology increases, the greater our interest in chloride becomes. In human studies, hyperchloremia has been identified as being able to reduce splanchnic blood flow, as measured by gastric tonometry [3]. Renal vasoconstriction has been induced experimentally by infusion of chloride-containing solutions in animal studies [4, 5]. In experimental sepsis, hyperchloremic acidosis provoked an increase in circulating inflammatory molecules [6]. Previous studies have reported an association between hyperchloremia and mortality in critically ill patients [7, 8], and in patients following non-cardiac surgery [9].

Severely injured trauma patients often initially require significant fluid resuscitation. According to Advanced Trauma Life Support guidelines, a bolus of 1–2 L of a warmed isotonic solution may be required to achieve an appropriate response in hemodynamically unstable patients [10]. The fluids most commonly used for resuscitation are 0.9 % normal saline (NS, 0.9 % NaCl), which contains supra-physiologic levels of sodium and chloride (154 mmol/L), and “balanced” crystalloids, such as Ringer’s lactate or Plasma-Lyte (Baxter Healthcare, Deerfield, IL, USA), which typically have more physiologic electrolyte concentrations (98–112 mmol/L chloride and 130–140 mmol/L sodium) [11]. Because it contains supra-physiologic levels of sodium and chloride, NS can cause hyperchloremic metabolic acidosis [12, 13]. As major trauma patients are commonly exposed to NS during the salvage phase of shock, they are susceptible to hyperchloremia in the post-resuscitation phase. The purpose of this study was to determine whether serum chloride levels are associated with mortality in patients who have sustained major trauma.

## Methods

### Study design and data collection

**Fig. 1:**
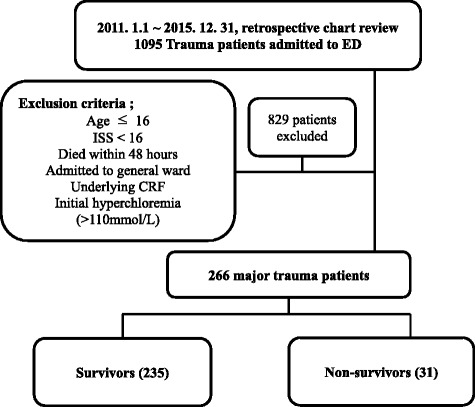
Study population by inclusion and exclusion criteria. ED, emergency department; ISS, injury severity score; CRF, chronic renal failure

We conducted a retrospective observational study in single center from January 2011 to December 2015. A total of 1095 trauma patients were admitted to the emergency department during this period. We excluded patients with the following characteristics: ≤16 years of age; injury severity score (ISS) < 16; admitted to a general ward; underlying chronic renal failure; died within 48 h of admission to the emergency department; and with initial hyperchloremia (chloride > 110 mmol/L). The patients with initial hyperchloremia (*n* = 18) had been transferred from other hospitals after receiving fluid resuscitation or other management. They were also transferred 3 days or more after injury. Consequently, 829 patients were excluded and 266 enrolled. Patients were divided into two groups: survivors (*n* = 235) and non-survivors (*n* = 31). These groups were compared with respect to clinical and biochemical variables (Fig. 1).

The study was approved by the Institutional Review Board (IRB No. 4-2016-0295), which waived the requirement for informed consent because of the retrospective nature of the study.

### Study variables and definition

Baseline characteristics included age, sex, underlying disease, Glasgow coma scale (GCS) score, and trauma related variables, such as ISS, revised trauma score (RTS), trauma and injury severity score (TRISS), mechanism(s) of injury and injury regions. Laboratory results were measured at initial presentation to the emergency unit at 48-h post-admission. Data regarding the length of hospital stay, length of intensive care unit stay and duration of mechanical ventilation were collected and analyzed.

Hyperchloremia was defined as a serum chloride level > 110 mmol/L. Delta chloride (Δchloride) was defined as the difference between the chloride level 48-h post-admission and the initial level. Body mass index was defined as body mass divided by the square of height (kg/m^2^), and GCS score refers to that obtained at the initial assessment. The Acute Physiology and Chronic Health Evaluation II (APACHE II) scores were calculated within the first day of ICU admission. Hypotension was defined as an initial systolic blood pressure < 90 mmHg, and hypothermia was defined as an initial body temperature < 35 °C. Emergency operations and emergency angio-embolization were defined as those performed within 24 h of admission. The total amount of fluid administered over 48 h was included, not only crystalloids but also colloids and blood transfusion products. NS infused over the first 48 h included fluid for resuscitation as well as that mixed with other drugs. Acute kidney injury (AKI) was defined according to the consensus Risk, Injury, Failure, Loss and End-Stage Renal Disease (RIFLE) definition [14] using serum creatinine changes with urine output criteria.

### Study outcomes

The primary outcome investigated in the multivariate logistic regression was 30-day hospital mortality, defined as all-cause mortality within 30-days of hospital admission.

### Statistical analysis

Statistical analysis was performed using IBM SPSS Statistics 20.0 (IBM Co. Armonk, NY). Categorical data were presented as number (%), and were compared using the chi-square or Fisher’s exact test. Continuous variables were expressed as the mean and standard deviation or median and inter-quartile range (IQR), and were compared between groups using the Student’s *t*-test or Mann-Whiney *U* test. Factors found to be significantly associated with mortality (*p*-value < 0.05) by univariate analysis were included in the multivariate analysis with age, as age is regarded as an important factor for mortality. APACHE II score was excluded from the analysis, because most of the variables that comprise the APACHE II score were included individually. Laboratory factors used in the analysis, such as hyperchloremia and base deficit, were the results at 48-h post-admission. Logistic regression analysis was performed independently to identify the association of 30-day mortality with hyperchloremia-48, absolute value of chloride and Δchloride by using the maximum likelihood method and backward stepwise selection. Goodness-of-fit was assessed using the Hosmer-Lemeshow test.

## Results

Of the 266 patients included in the study, 235 (88 %) survived and 31 (12 %) died within 30 days of admission. The mean age of the patients was 49.7 years and 196 (74 %) patients were male with no significant difference between survivors and non-survivors in terms of age and sex. Mean ISS, RTS and APACHE II scores differed significantly between the two groups (Table 1).

**Table 1:** Baseline characteristics of major trauma patients

	Total (266)	Survivors (235)	Non-survivors (31)	*p* value
Age (years), mean ± SD	49.7 ± 19.5	49.1 ± 19.7	54.2 ± 17.6	0.17
Male, *n* (%)	196 (74)	173 (74)	23 (74)	1.00
Underlying disease, *n* (%)				
HTN	50 (19)	42 (18)	8 (26)	0.33
DM	32 (12)	27 (12)	5 (16)	0.55
Pulmonary tuberculosis	4 (2)	3 (1)	1 (3)	0.39
Hepatitis	3 (1)	2 (1)	1 (3)	0.31
BMI, mean ± SD	23.2 ± 3.0	23.2 ± 3.0	23.7 ± 2.9	0.36
GCS, median (IQR)	15.0 (7.0–15.0)	15.0 (9.0–15.0)	5.0 (3.0–12.0)	<0.001
ISS, mean ± SD	26.0 ± 7.9	25.4 ± 7.6	30.8 ± 8.4	<0.001
RTS, mean ± SD	6.726 ± 1.684	6.939 ± 1.465	5.112 ± 2.298	<0.001
TRISS, mean ± SD	79.1 ± 26.8	83.7 ± 23.0	47.0 ± 32.2	<0.001
APACHE II, mean ± SD	16.2 ± 9.2	14.7 ± 8.3	27.1 ± 8.5	<0.001
Injury mechanism, *n* (%)				0.26
MVA (pedestrian)	86 (32)	73 (31)	13 (42)	
MVA (passenger)	39 (15)	38 (16)	1 (3)	
Motorcycle accidents	58 (22)	53 (23)	5 (16)	
Falls	63 (24)	53 (23)	10 (32)	
Penetrating injuries	5 (2)	5 (2)	0 (0)	
Others	15 (6)	13 (6)	2 (7)	
Injury region, *n* (%)				
Head & Neck	209 (79)	182 (77)	27 (87)	0.25
Face	105 (40)	94 (40)	11 (36)	0.70
Chest	188 (71)	172 (73)	16 (52)	0.02
Abdomen	143 (54)	127 (54)	16 (52)	0.85
Extremities	179 (67)	160 (68)	19 (61)	0.54
External	193 (73)	171 (73)	22 (71)	1.00

*SD* standard deviation, *DM* diabetes mellitus, *BMI* body mass index, *GCS* Glasgow coma scale, *ISS* injury severity score, *RTS* revised trauma score, *TRISS*, trauma and injury severity score, *APACHE II* acute physiology and chronic health evaluation II, *MVA* motor vehicle accidents

Hypotension and hypothermia were detected in 22 (71 %) and 10 (32 %) non-survivors, respectively. Both these proportions were significantly greater compared to those of survivors (Table 2). There was a significant difference between survivors and non-survivors in terms of receiving blood-product transfusions (50 % vs. 87 %, respectively, *p* < 0.001). The median volume of fluid infused during the first 48 h was also significantly different between survivors and non-survivors (7.4 L vs. 11.8 L, *p* < 0.001), but volumes of NS infused were not significantly different (2.0 L vs. 2.1 L, *p* = 0.75). Overall, the median positive fluid balance was 2.2 L, significantly higher in non-survivors than in survivors (2.1 L vs. 2.7 L cumulative fluid balance at 48 h, *p* = 0.03).

**Table 2:** Comparison of clinical parameters between survivors and non-survivors

	Total (266)	Survivors (235)	Non-survivors (31)	*p* value
Hypotension (SBP < 90), *n* (%)	105 (40)	83 (35)	22 (71)	<0.001
Hypothermia (BT < 35°C), *n* (%)	36 (13)	26 (11)	10 (32)	0.003
Drug exposure during 48 h, *n* (%)				
Vasopressor	107 (40)	78 (33)	29 (94)	<0.001
Loop diuretics	123 (46)	101 (43)	22 (71)	0.004
NSAIDs	46 (17)	43 (18)	3 (10)	0.32
Aminoglycoside	27 (10)	25 (11)	2 (7)	0.55
Transfusion, *n* (%)	144 (54)	117 (50)	27 (87)	<0.001
Total volume of infused fluid during 48 h (L), median (IQR)	7.6 (6.3–10.1)	7.4 (6.2–9.3)	11.8 (8.2–14.6)	<0.001
Total volume of infused NS during 48 h (L), median (IQR)	2.0 (1.2–3.2)	2.0 (1.2–3.2)	2.1 (1.3–3.2)	0.75
Total volume of infused Plasmalyte during 48 h (L), median (IQR)	2.0 (1.0–3.0)	2.0 (1.0–3.0)	3.0 (1.0–3.6)	0.22
Cumulative fluid balance at 48 h (L), median (IQR)	2.2 (1.2–4.0)	2.1 (1.1–3.7)	2.7 (1.8–6.8)	0.03

*SBP* systolic blood pressure, *BT* body temperature, *IQR* interquartile range, *NSAIDs* nonsteroidal anti-inflammatory drugs

There were no significant differences in electrolyte levels at initial measurement. However, 48 h post-admission, sodium (140.6 ± 4.31 mmol/L vs. 148.5 ± 10.1 mmol/L, *p* < 0.001) and chloride (105.7 ± 4.7 mmol/L vs. 112.6 ± 10.7 mmol/L, *p* = 0.002) levels were significantly higher in non-survivors than in survivors, respectively. Base deficit and lactate levels also differed significantly between the two groups. The difference in chloride level between the 48-h post-admission and initial measurement, Δchloride, was significantly higher in non-survivors (10.3 ± 11.1 mmol/L) than in survivors (1.7 ± 5.2 mmol/L, *p* < 0.001). In addition, increases in Δchloride were significantly different between the two groups (Table 3).

**Table 3:** Comparison of laboratory findings between survivors and non-survivors

	Total (266)	Survivors (235)	Non-survivors (31)	*p* value
Creatinine (μmol/L), mean ± SD				
Initial	79.6 ± 38.9	79.6 ± 39.8	88.4 ± 25.6	0.20
At 48 h.	79.6 ± 21.2	79.6 ± 39.8	132.6 ± 98.1	0.001
Sodium (mmol/L), mean ± SD				
Initial	140.6 ± 2.9	140.6 ± 2.7	140.6 ± 3.5	0.93
At 48 h	141.5 ± 5.8	140.6 ± 4.3	148.5 ± 10.1	<0.001
Potassium (mmol/L), mean ± SD				
Initial	3.9 ± 0.5	3.9 ± 0.5	4.0 ± 0.6	0.63
At 48 h	3.8 ± 0.5	3.8 ± 0.4	3.8 ± 0.8	0.84
Chloride (mmol/L), mean ± SD				
Initial	103.9 ± 3.3	104.1 ± 3.2	102.3 ± 3.9	0.006
At 48 h	106.5 ± 6.1	105.7 ± 4.7	112.6 ± 10.7	0.002
Δchloride (mmol/L), mean ± SD	2.6 ± 6.6	1.7 ± 5.2	10.3 ± 11.1	<0.001
Δchloride (mmol/L), *n* (%)				<0.001
-10 ≤ < 0	80 (32)	77 (34)	3 (11)	
0 ≤ <10	148 (59)	134 (60)	14 (52)	
10 ≤ <20	18 (7)	12 (5)	6 (2)	
20 ≤ <30	5 (2)	2 (1)	3 (11)	
40 ≤	1 (1)	0 (0)	1 (4)	
pH, mean ± SD				
Initial	7.36 ± 0.10	7.37 ± 0.88	7.32 ± 0.18	0.16
At 48h	7.50 ± 1.33	7.52 ± 1.43	7.37 ± 0.09	0.59
Base deficit (mmol/L), mean ± SD				
Initial	−5.7 ± 4.0	−5.4 ± 3.8	−8.2 ± 4.6	0.003
At 48 h	−1.0 ± 3.0	−0.6 ± 2.7	−3.4 ± 4.1	<0.001
Lactate (mmol/L), mean ± SD				
Initial	3.9 ± 3.1	3.6 ± 2.7	6.4 ± 4.4	0.003
At 48 h	1.8 ± 1.6	1.5 ± 1.3	3.4 ± 2.9	0.03

*SD* standard deviation

The median overall length of hospital stay was longer for survivors (25.0 days) than non-survivors (8.0 days, *p* < 0.001), although there was no difference in the median duration of ICU stay. While emergency operations were more frequently performed in non-survivors, there was no difference between the groups in the proportion of patients undergoing angio-embolization. Hyperchloremia 48-h post-admission (hyperchloremia-48) was more common in non-survivors (Table 4).

**Table 4:** Clinical outcomes of survivors and non-survivors

	Total (266)	Survivors (235)	Non-survivors (31)	*p* value
LoH (day), median (IQR)	22.0 (12.0–48.3)	25.0 (15.0–54.0)	8.0 (4.0–15.5)	<0.001
LoICU (day), median (IQR)	5.5 (3.0–14.0)	5.0 (3.0–14.0)	6.0 (4.0–14.0)	0.36
Mechanical Ventilation, *n* (%)	166 (62)	136 (58)	30 (97)	<0.001
DoMV (day), median (IQR)	3.0 (0.0–9.0)	2.0 (0.0–9.0)	6.0 (3.0–14.0)	0.001
Emergency operation, *n* (%)	92 (35)	79 (34)	18 (58)	0.01
Angioembolization, *n* (%)	57 (21)	48 (20)	9 (29)	0.35
AKI, *n* (%)	82 (31)	60 (26)	22 (71)	<0.001
Hyperchloremia-48, *n* (%)	65 (24)	46 (20)	19 (61)	<0.001

*LoH* length of hospital stay, *IQR* interquartile range, *LoICU* length of intensive care unit stay, *DoMV* duration of mechanical ventilation, *AKI* acute kidney injury, *Hyperchloremia-48* hyperchloremia 48-h post-admission

Multivariate analysis revealed that hyperchloremia-48 (adjusted odds ratio [OR] = 4.567, 95 % confidence interval [CI] = 1.634–12.764, *p* = 0.004), RTS, base deficit, use of vasopressor and AKI were independent predictors for 30-day mortality in major trauma patients (Table 5). In addition, the absolute value of chloride at 48 h (OR = 1.075, 95 % CI = 1.006–1.150, *p* = 0.03, Table 6) and Δchloride (OR = 1.096, 95 % CI = 1.027–1.169, *p* = 0.006, Table 7) were independent risk factors for 30-day mortality in major trauma patients.

**Table 5:** Univariate and multivariate analyses evaluating the association of hyperchloremia-48 on 30-day mortality

Variables	Univariate analysis		Multivariate analysis	
	OR (95 % CI)	*p* value	OR (95 % CI)	*p* value
Age (per year)	1.014 (0.994–1.034)	0.17	.	
ISS	1.080 (1.033–1.130)	0.001	.	
RTS	0.621 (0.511–0.754)	<0.001	0.715 (0.551–0.929)	0.01
Mechanical ventilation (yes)	21.838 (2.929–162.842)	0.003	.	
Transfusion (yes)	6.750 (2.290–19.894)	0.001	.	
Hypotension (SBP < 90, yes)	4.477 (1.971–10.168)	<0.001	.	
Hypothermia (BT < 35 °C, yes)	3.828 (1.626–9.012)	0.002	.	
Cumulative fluid balance at 48 h (per L)	1.000 (1.000–1.000)^a^	0.01	.	
Base deficit at 48 h (per 1mmol/L)	0.740 (0.631–0.868)	<0.001	0.782 (0.650–0.940)	0.009
Hyperchloremia-48 (yes)	8.667 (3.736–20.103)	<0.001	4.567 (1.634–12.764)	0.004
Use of vasopressor (yes)	29.186 (6.789–125.472)	<0.001	9.233 (1.925–44.295)	0.005
Use of loop diuretics (yes)	3.243 (1.432–7.344)	0.005	.	
AKI (yes)	7.130 (3.112–16.336)	<0.001	2.574 (0.650–0.940)	0.08

*OR* odds ratio, *CI* confidence interval, *ISS* injury severity score, *RTS* revised trauma score, *AKI* acute kidney injury, *SBP* systolic blood pressure, *BT* body temperature, *AKI* acute kidney injury

^a^(1.000027–1.000216)

*p* value for the Hosmer-Lemeshow goodness-of-fit-test was 0.256

**Table 6:** Univariate and multivariate analyses evaluating the association of absolute value of Chloride (mmol/L) on 30-day mortality

Variables	Univariate analysis		Multivariate analysis	
	OR (95 % CI)	*p* value	OR (95 % CI)	*p* value
Age (per year)	1.014 (0.994–1.034)	0.17	.	
ISS	1.080 (1.033–1.130)	0.001	.	
RTS	0.621 (0.511–0.754)	<0.001	0.720 (0.552–0.939)	0.02
Mechanical ventilation (yes)	21.838 (2.929–162.842)	0.003	.	
Transfusion (yes)	6.750 (2.290–19.894)	0.001	3.296 (0.756–14.372)	0.11
Hypotension (SBP < 90, yes)	4.477 (1.971–10.168)	<0.001	.	
Hypothermia (BT < 35 °C, yes)	3.828 (1.626–9.012)	0.002	.	
Cumulative fluid balance at 48 h (per 1L)	1.000 (1.000–1.000)^a^	0.01	.	
Base deficit at 48 h (per 1mmol/L)	0.740 (0.631–0.868)	<0.001	0.748 (0.619–0.905)	0.003
Chloride at 48 h (per 1mmol/L)	1.151 (1.084–1.222)	<0.001	1.075 (1.006–1.150)	0.03
Use of vasopressor (yes)	29.186 (6.789–125.472)	<0.001	9.758 (2.044–46.600)	0.004
Use of loop diuretics (yes)	3.243 (1.432–7.344)	0.005	.	
AKI (yes)	7.130 (3.112–16.336)	<0.001	.	.

*OR* odds ratio, *CI* confidence interval, *ISS* injury severity score, *RTS* revised trauma score, *AKI* acute kidney injury, *SBP* systolic blood pressure, *BT* body temperature, *AKI* acute kidney injury

^a^(1.000027–1.000216)

*p* value for the Hosmer-Lemeshow goodness-of-fit-test was 0.148

**Table 7:** Univariate and multivariate analyses evaluating the association of Δchloride (mmol/L) on 30-day mortality

Variables	Univariate analysis		Multivariate analysis	
	OR (95 % CI)	*p* value	OR (95 % CI)	*p* value
Age (per year)	1.014 (0.994–1.034)	0.17	.	
ISS	1.080 (1.033–1.130)	0.001	.	
RTS	0.621 (0.511–0.754)	<0.001	0.728 (0.559–0.950)	0.02
Mechanical ventilation (yes)	21.838 (2.929–162.842)	0.003	.	
Transfusion (yes)	6.750 (2.290–19.894)	0.001	.	
Hypotension (SBP < 90mmHg, yes)	4.477 (1.971–10.168)	<0.001	.	
Hypothermia (BT < 35 °C, yes)	3.828 (1.626–9.012)	0.002	.	
Cumulative fluid balance at 48 h (per L)	1.000 (1.000–1.000)^a^	0.01	.	
Base deficit at 48 h (per 1 mmol/L)	0.740 (0.631–0.868)	<0.001	0.761 (0.631–0.918)	0.004
Δchloride (per 1 mmol/L)	1.165 (1.096–1.238)	<0.001	1.096 (1.027–1.169)	0.006
Use of vasopressor (yes)	29.186 (6.789–125.472)	<0.001	10.392 (2.222–48.604)	0.003
Use of loop diuretics (yes)	3.243 (1.432–7.344)	0.005	.	
AKI (yes)	7.130 (3.112–16.336)	<0.001	.	

*OR* odds ratio, *CI* confidence interval, *ISS* injury severity score, *RTS* revised trauma score, *AKI* acute kidney injury, *SBP* systolic blood pressure, *BT* body temperature, *AKI* acute kidney injury

^a^(1.000027–1.000216)

*p* value for the Hosmer-Lemeshow goodness-of-fit-test was 0.835

## Discussion

This study comprised seriously injured patients (ISS ≥ 16) admitted to a trauma ICU. Hyperchloremia occurred frequently in patients who had normal levels of serum chloride on admission; 24 % had serum chloride levels > 110 mmol/L 48-h post-admission. Multivariate logistic regression analysis identified hyperchloremia-48 and Δchloride as being independently associated with 30-day mortality, adjusted for other significant variables. Similar to previous studies that reported an association between hyperchloremia and mortality in critically ill [7, 8], and postoperative patients [9], our study showed that elevated chloride levels may be associated with mortality in major trauma patients.

Pro-inflammatory responses to hyperchloremic metabolic acidosis are mediated by nitric oxide and are associated with a higher interleukin (IL)-6 to IL-10 ratio when compared with lactic acidosis, as reported in in vitro cell models [15]. Hyperchloremic metabolic acidosis may be a pro-inflammatory modulator in sepsis [6, 16, 17]. Neutrophil function is also influenced by chloride influx through different chloride channels and via cotransporters [18, 19]. Low or absent extracellular chloride concentrations are associated with decreased neutrophil function [20, 21].

Associations between fluid overload and morbidity and mortality were reported by the Fluid and Catheter Treatment Trial (FACTT) and other trials in surgical patients [22–24]. When administered in large volumes, NS has been shown to cause coagulopathy [25, 26]. The mechanisms underlying saline-induced coagulopathy are not fully known. Our results showed that non-survivors had significantly higher total volumes of fluid infused compared to survivors (7.4L vs. 11.8L). However, these patients could have had greater fluid demands because of hypotension or the need to undergo emergency operations (which was significantly higher in non-survivors than in survivors). Shaw et al. [11] revealed that resuscitation volumes ≤ 1500 mL, which may reflect a low total chloride load (0–300 mmol), were not associated with increased mortality. However, there was a trend towards increasing mortality with increasing chloride load in patients receiving ≥ 1500 mL of fluid for resuscitation, which may reflect the potential risk of resuscitation with chloride-rich fluids. In the present study, the overall median total fluid infusion volume was 7.6 L (IQR 6.3–10.1 L) over 48 h. Total chloride loads may have been enough to show this trend, but we were unable to calculate the volume-adjusted chloride load. Some data were missing and we could not include other kinds of infused fluid, such as blood transfusion products and colloid.

Infusion of chloride-rich solutions, such as NS, is itself associated with hyperchloremia [1, 13, 27–29] which has been associated with poor patient outcomes [8, 9]. Shaw et al. [30] considered postoperative outcomes following abdominal surgery: Their analysis used propensity score matching to match 926 patients receiving Plasma-Lyte in a 3:1 ratio to patients receiving NS. Patients receiving NS were more likely to suffer major postoperative complications, AKI and infection. Similarly, large changes in serum chloride levels correlated with greater in-hospital mortality [11]. However, a meta-analysis of high-versus low-chloride content in perioperative and critical care fluid resuscitation concluded that a weak but significant association of unfavorable outcomes was found, but that mortality was unaffected by chloride content [31]. Our results showed significant differences between survivors and non-survivors with the groups of Δchloride. These findings contribute to the evidence of the potentially serious clinical impact of using chloride-rich crystalloids that could induce hyperchloremia.

The present study has a number of limitations. First, this was a retrospective, single tertiary center study. Second, the fluid resuscitation strategy could not be controlled because of retrospective data collection. Similarly, pre-hospital fluid resuscitation was not included. However, we did exclude patients with hyperchloremia at initial measurement on admission. Third, as mentioned previously, we were unable to include other kinds of infused fluid, such as blood products and colloids. We were also unable to calculate the volume-adjusted chloride load. Fourth, selection bias may have been possible given the inclusion and exclusion criteria. Lastly, because of the limited patient numbers, our results are not representative of all major trauma patients. Larger, controlled studies are need for greater power and reliability.

## Conclusion

Hyperchloremia 48-h post-admission occurs frequently in patients who have sustained major trauma. We found that hyperchloremia-48 and Δchloride were independently associated with all cause 30-day mortality in major trauma patients. These variables might be useful to predict mortality in patients who have sustained major trauma. However, data from large, prospective, controlled studies that are adequately powered are needed to detect differences in outcomes such as mortality and morbidity.

## Abbreviations

AKI: Acute kidney injury
APACHE II: Acute Physiology and Chronic Health Evaluation II
CI: Confidence interval
GCS: Glasgow coma scale
ICU: Intensive care unit
IL: Interleukin
IQR: Inter-quartile range
ISS: Injury severity score
NS: Normal saline
OR: Odds ratio
RTS: Revised trauma score
TRISS: Trauma and injury severity score
